# A Step Forward in Long COVID Research: Validating the Post-COVID Cognitive Impairment Scale

**DOI:** 10.3390/ejihpe14120197

**Published:** 2024-12-01

**Authors:** Somayeh Pour Mohammadi, Razieh Etesamipour, Francisco Mercado Romero, Irene Peláez

**Affiliations:** 1 Department of Psychology, School of Health Sciences, Rey Juan Carlos University, 28922 Madrid, Spain; s.pour.2020@alumnos.urjc.es (S.P.M.); francisco.mercado@urjc.es (F.M.R.); 2 Department of Psychology, Payame Noor University (PNU), Tehran 19395-4697, Iran; r_etesamipour@pnu.ac.ir

**Keywords:** long COVID, cognitive impairment, post-COVID syndrome, memory, attention, neuro-long COVID

## Abstract

Long COVID, or post-acute sequelae of SARS-CoV-2 infection, includes a variety of enduring symptoms that endure beyond the acute phase of the illness, impacting multiple facets of patients’ psychological and physical health. The persistent symptoms encompass fatigue, breathing difficulties, musculoskeletal pain, and cognitive impairments, which can significantly affect daily functioning and overall quality of life. The objective of this study was to create and validate the accuracy of the Post-COVID Cognitive Impairment Scale, which is used to evaluate cognitive impairments resulting from a COVID-19 infection. This study was conducted in Iran between January and September 2023. It consisted of three phases: developing the scale, evaluating its content validity with experts, and validating its structure with 454 participants using exploratory and confirmatory factor analysis. The exploratory factor analysis revealed two variables, namely memory and attention, which accounted for 40.38% of the variation. Confirmatory factor analysis verified the model’s fit, with indices indicating satisfactory alignment: CMIN/DF = 2.80, RMSEA = 0.06, SRMR = 0.05, CFI = 0.93, and TLI = 0.92. The factor loadings were statistically significant (*p* < 0.001), and Cronbach’s Alpha values indicated strong internal consistency (working memory = 0.81, attention = 0.80). These results affirm the Post-COVID Cognitive Impairment Scale is a valid and reliable instrument for evaluating cognitive deficiencies in individuals with long COVID. Its application in clinical and research environments aids in the prompt detection and tracking of the treatment of such impairments.

## 1. Introduction

After the pandemic had been going on for more than three years, the Emergency Committee on COVID-19 of the World Health Organization (WHO) submitted a recommendation in May 2023 stating that the pandemic was no longer considered a public health emergency of international concern [1]. Although the acute phase of COVID-19 has decreased, the persistent consequences of the virus are still ongoing. A study found that 68.7% of non-hospitalized COVID-19 patients experienced post-acute sequelae within 30 days of infection, with some symptoms persisting for 12 weeks or more [2,3,4].

The enduring ramifications of SARS-CoV-2 infection are becoming increasingly concerning.

A considerable proportion of patients who have recuperated from the virus encounter enduring symptoms, a condition now often referred to as “long COVID.” The persistent symptoms, known as “post-acute COVID-19”, “post-COVID-19 syndrome”, or “post-COVID-19 condition”, have been clinically termed “post-acute sequelae of COVID-19”, (PASC) [5] by the National Institutes of Health. This condition provides a new public health challenge that requires immediate attention and comprehensive understanding [6,7,8].

The most debilitating symptoms associated with long COVID include fatigue [9,10], cognitive impairments—commonly known as “brain fog” [11,12], which typically manifest as difficulties with memory, attention, concentration, and multitasking [13]—headache, and insomnia, all of which significantly impair daily activities [14,15]. The symptoms, termed neuro-long COVID [16,17], affect millions globally and frequently manifest in people with moderate initial COVID-19 cases who did not necessitate hospitalization with pneumonia or hypoxemia [18,19].

This pervasive problem has had a substantial impact on people’s lives and poses a challenge to the conventional view of recuperation after an illness. As the medical community strives to comprehend the full extent of chronic COVID-19, the urgent inquiry persists: How can we efficiently monitor and control these persistent symptoms?

### 1.1. Manifestations of Long COVID and Neuro-Long COVID

Long COVID includes a variety of enduring symptoms that can impact several organ systems, with neurological manifestations being especially significant [20,21]. These symptoms endure following the clearance of the acute infection and may vary from moderate to severe, persisting for months and perhaps leading to the emergence of additional symptoms post-infection [22].

In order to accurately monitor changes in symptoms among patients with COVID-19, several nations have implemented specialized tools. Participants in the UK survey conducted by the Office for National Statistics reported experiencing any of the following 12 symptoms in the past seven days: fever, headache, muscle ache, weakness/tiredness, nausea/vomiting, abdominal pain, diarrhea, sore throat, cough, shortness of breath, loss of taste, and loss of smell [23,24]. Similar questions were asked of participants in the Understanding America Study—COVID-19 Survey regarding symptoms like fever or chills, runny or stuffy nose, chest congestion, cough, sore throat, sneezing, headaches, muscle or body aches, fatigue, shortness of breath, abdominal pain, body temperature above 100.4 °F or 38.0 °C, vomiting, hair loss, dry skin, diarrhea, loss of smell, and skin rash [25].

In March 2023, the Office for National Statistics in the UK revealed that 1.5 million individuals (79% of those with self-reported long COVID) experienced negative effects on their daily activities due to symptoms. Among them, 381,000 individuals (20%) reported significant limitations in their ability to carry out their day-to-day tasks. The predominant symptom described by persons experiencing long COVID was fatigue, with 72% of those self-reporting long COVID experiencing this symptom. This was followed by difficulty concentrating (51%), muscle aches (49%), and shortness of breath (48%) [26].

In 2021, the National Institutes of Health (NIH), alongside multicenter studies in the United States and international research, established that these symptoms are more prevalent in patients with a history of severe illness [7,27,28]. However, findings also suggest that long COVID can manifest in young individuals, children, and patients with mild COVID-19 symptoms who did not necessitate hospitalization or respiratory support [29,30,31].

Beyond physiological symptoms, various research has examined the neurological and cognitive manifestations of long COVID, commonly termed neuro-long COVID [5]. The results of this research have resulted in the identification of the subsequent symptoms:

- Mood and psychological symptoms: Patients with long COVID frequently have feelings of sadness, anxiety [32], depression [33], and post-traumatic stress disorder [34]. These symptoms correlate with diminished quality of life and cognitive performance, highlighting the necessity for emotional support and focused intervention [35];
- Neurological examination findings: Neurological consequences are progressively acknowledged in individuals recovering from COVID-19, involving a wide array of symptoms and syndromes [36,37,38]. Abnormal neurological findings, such as numbness or tingling sensations, are more prevalent in post-hospitalized patients compared to non-hospitalized persons [37]. Frequent neurological symptoms encompass headache, fatigue, dizziness, anosmia, ageusia, anorexia, and myalgias [36,38]. Severe instances of COVID-19 have been linked to more grave neurological disorders, including meningoencephalitis, intracerebral hemorrhage, altered consciousness, syncope, seizures, and stroke [36];
- Chronic fatigue syndrome [39], pain in the muscles, and headaches are prevalent symptoms that intersect with neurological signs, hence complicating the clinical presentation of long COVID [40]. Chronic headaches are commonly documented, adding to the total symptomatology [35];
- Sleep disorders: Individuals with long COVID usually experience sleep disorders, such as insomnia, which contribute to overall fatigue and cognitive deficits [41];
- Cognitive impairments: Cognitive deficiencies have emerged as a primary symptom linked to neuro-long COVID, impacting all facets of cognitive function in a varied and frequently overlapping manner [11,17]. While research in this domain is ongoing, data indicates that cognitive difficulties, including memory impairment, concentration problems, and “brain fog”, are among the most commonly reported symptoms, affecting around 70% of persons [12,42,43]. In a 7-month research cohort, 85.1% of participants (3203 individuals) reported having brain fog and cognitive impairment, encompassing deficits in attention, executive function, problem-solving, and decision-making [12]. In another study, 86% of participants indicated that post-COVID-19 cognitive impairment substantially impacted their every day work capabilities [11,12]. Similarly, another study identified prevalent impairments such as trouble concentrating (77.8%), brain fog (69%), forgetfulness (67.5%), tip-of-the-tongue word retrieval difficulties (59.5%), and semantic disfluency (43.7%) [12]. Research demonstrates that a considerable percentage of persons with long COVID exhibit cognitive impairments in at least one area, predominantly affecting executive functions [35,41].

Cognitive symptoms differ among individuals and frequently impact various domains, including memory (working memory, retrospective, and prospective), attention, language, and executive skills, all of which may have interrelated implications on everyday functioning [11]. Memory deficits can limit the capacity to create new memories, retrieve prior information, and retain information temporarily for processing tasks, hence influencing working memory as well as retrospective and prospective memory [44]. Likewise, attention-related deficiencies may present as difficulties in maintaining focus, selective or divided attention [44], and shifting attention between tasks. Language and executive functions [11,35,41] may be impaired, manifesting as word-finding difficulties, diminished planning and problem-solving abilities, and reduced cognitive flexibility, thereby affecting the capacity to adjust to new information or evolving situations.

### 1.2. Assessment Tools for Long COVID Symptoms

The evaluation instruments for symptoms of long COVID have attracted interest for their capacity to encapsulate the intricate interaction of psychological, cognitive, and physical symptoms. Hughes and co-workers [45] developed a questionnaire to evaluate the intensity of symptoms in persons suffering with long COVID. The researchers utilized Rasch analysis and incorporated 131 items that were categorized into 17 scales, including a wide range of symptoms. Bahmer and co-workers [46] devised a metric in Germany to evaluate the post-COVID condition, with a special focus on the physical repercussions in various organs and systems. The researchers utilized k-means clustering and ordinal logistic regression analysis to assess the intensity of symptoms. Yuan Kuo and co-workers [47] assessed the severity of long COVID symptoms using a 24-item scale, emphasizing the continued presence of both physical and psychological symptoms for a minimum of three months following a COVID-19 infection. Several studies have recorded the presence of sadness, cognitive impairments, and sleep disturbances in persons experiencing long COVID [2,47,48,49].

The Neurobehavioral Symptom Inventory (NSI) is also a commonly employed tool that proficiently assesses neurobehavioral symptoms in affected persons [50]. The Neurobehavioral Symptom Inventory (NSI) is a 22-item self-report instrument designed to assess somatosensory, cognitive, and affective symptoms [50]. Nonetheless, although the NSI offers significant insights, it may not entirely capture the range of cognitive impairments experienced by patients, including focus and memory difficulties, which are common in this demographic [51,52]. The Symptom Burden Questionnaire for Long COVID (SBQ-LC) is a validated patient-reported outcome measure developed using psychometric approaches. This questionnaire seeks to thoroughly evaluate the symptom burden encountered by patients, encompassing neurocognitive symptoms such as memory impairment and attentional challenges [45]. The development of these tools is based on patients’ life experiences, hence increasing their relevance and application in clinical environments. Notwithstanding these advantages, the reliability of these assessment instruments remains a significant issue. The long COVID symptom and impact tools have been evaluated for test-retest reliability, demonstrating a level of stability over time [53]. However, the subjective aspect of patient-reported outcomes can add variability, especially in neurocognitive symptoms that may fluctuate due to several causes, including exhaustion and emotional condition. Furthermore, the notion of a “patient-acceptable symptomatic state” (PASS) has been created to delineate thresholds for tolerable symptom levels. This is particularly significant for neurocognitive symptoms, as numerous patients indicate an intolerable symptomatic condition, complicating the interpretation of evaluation outcomes [54]. The PASS score for the long COVID impact tool suggests that a considerable percentage of patients endure incapacitating symptoms, which may not be entirely reflected by current assessment instruments. Construct validity represents an additional area of concern. The correlation between the scores of these instruments and health-related quality of life has been evaluated, although the particular subtleties of neurocognitive symptoms may not be sufficiently captured [11,55,56].

Patient-reported outcome measures (PROMs) are crucial for documenting the experiences of patients with long COVID, offering insights into the effects of symptoms on everyday activities [57,58]. Nonetheless, dependence on self-reported data can create biases, as patients may either underreport or overreport symptoms influenced by their emotional state or comprehension of their situation. 

Furthermore, functional MRI (fMRI) investigations have elucidated changes in brain connectivity and activity during cognitive tasks in patients with long COVID, emphasizing the neurological basis of their symptoms [59,60]. Despite its efficacy, the prohibitive cost and restricted accessibility of fMRI may impede its extensive utilization in clinical environments.

Another method for evaluating cognitive domains in long COVID involves the utilization of a thorough Neuropsychological Test Battery [42]. This battery includes various cognitive domains and utilizes a variety of neuropsychological assessments administered directly by qualified neuropsychologists in face-to-face sessions rather than through self-report questionnaires. The assessments, including the Wechsler Memory Scale (WMS-IV), Stroop Test, Trail Making Test [61], Number Span, and Hopkins Verbal Learning Test–Revised [62], offer a more precise and sophisticated evaluation of cognitive capabilities. All of them highlighted the significant influence of COVID-19 on cognitive abilities [48,49,61,62,63].

The findings demonstrated that individuals experiencing neurological symptoms such as headache, loss of smell, and altered taste, as well as those who were hospitalized or needed oxygen therapy, showed reduced cognitive performance in these specific areas. This establishes a definitive connection between COVID-19 infection and cognitive impairments [61,62]. ICU patients demonstrated more severe and extensive cognitive deficits [64]. Nonetheless, the resource-demanding characteristics of these evaluations, frequently extending over several hours, may restrict their practicality in standard clinical environments.

### 1.3. The Importance of Questionnaires in Assessing Cognitive Impairments in Long COVID

Despite prior research providing significant insights into the neurocognitive difficulties associated with long COVID, a substantial gap persists in the literature concerning specialized instruments explicitly developed to evaluate cognitive impairments in this demographic. The majority of cognitive assessments performed during the pandemic predominantly utilized neuropsychological tests conducted in controlled environments rather than questionnaires designed for real-world circumstances [12,44,61,62,65,66]. Conventional tests, although comprehensive, frequently do not reflect the dynamic and situational intricacies of cognitive impairment encountered in the daily lives of patients with long COVID [17,44].

Technological advancements have facilitated the development of advanced technologies that can identify structural and functional elements of neurological abnormalities [67]. Nonetheless, these instruments are not consistently available or feasible for extensive screening and may fail to accurately represent the cognitive experiences of individuals with long COVID. Moreover, current questionnaire-based assessments typically amalgamate somatic and psychological symptoms [45,46,47,49], placing little attention on cognitive deficits as a distinct area of concern. The absence of specificity hinders their ability to comprehend and address the distinct cognitive challenges faced by patients with long COVID.

Questionnaires provide a distinctive and vital function as accessible, ecological, and pragmatic instruments in cognitive evaluation [68]. Their simplicity, versatility, cost-effectiveness, and efficiency render them indispensable for extensive applications, particularly for Long COVID. Screening questionnaires tailored for cognitive evaluation can enhance accessibility, enabling the identification and referral of individuals who might otherwise go undetected [69]. This is especially pertinent for individuals in diverse environments, as questionnaires can provide a more comprehensive representation of cognitive deficits that may be overlooked in laboratory tests.

Moreover, cognitive assessment questionnaires are based on patients’ daily experiences, highlighting the manifestation of cognitive problems in real-world contexts. This method is particularly beneficial for patients with long COVID, who frequently experience challenges with memory, attention, and problem-solving in their daily tasks. By emphasizing daily functionality, these assessments offer a significant assessment of how cognitive impairments influence patients’ quality of life and their capacity to engage with their environment [70]. In settings characterized by elevated cognitive demands or intricate stimuli, individuals with cognitive impairments may experience exacerbated difficulties, impacting their overall productivity and social interaction [71]. 

At present, there is no questionnaire particularly validated for post-COVID cognitive impairment that possesses both known reliability and validity. This gap highlights the urgent necessity for a technique that can precisely measure cognitive alterations in patients with long COVID, as cognitive function is essential to practically all facets of daily life, ranging from basic chores to intricate problem-solving.

### 1.4. Objective of the Study

Given this background, our objective was to formulate and validate a set of patient-reported instruments for monitoring cognitive impairment following the acute phase of COVID-19. The Post-COVID-19 Cognitive Impairment Scale refers to cognitive deficits that arise in persons with Long COVID. These cognitive deficits often continue for 6 months after the initial onset of COVID-19 and cannot be attributed to any other medical conditions.

## 2. Materials and Methods

### 2.1. Study Design

The study employed a cross-sectional, descriptive, exploratory research approach to create the novel Post-COVID-19 Cognitive Impairment Scale. This scale was specifically designed to assess the severity of cognitive impairment symptoms among people with a previous diagnosis of COVID-19. 

The scale underwent development and validation in Iran in a three-phase procedure spanning from January 2023 to September 2023.

### 2.2. Instrument and Procedure

- Phase I: Formulation of the Scale (5 January 2023 to 14 March 2023).

Through a literature review, we identified two cognitive components (attention and memory) affected by COVID-19 [45,63,64,66].

To develop the questionnaire, we gathered lists of daily tasks that need the cognitive ability specified. 

Subsequently, test questions were created to measure everyday activities in each cognitive domain. For this situation, the questionnaire comprised a set of questions for each design component, with seven to ten questions for each. Ultimately, 16 questions were chosen from the available items. The questionnaire had 16 items that evaluated cognitive ability in everyday situations.

Participants evaluated each topic using a five-point Likert scale, with “very little” (score 1) indicating minimal impairment and “very much” (score 5) indicating significant impairment. A higher scale score indicates a more pronounced level of symptom intensity.

- Phase II: Evaluation of Content Validity (15 March 2023 to 25 April 2023).

Five psychologists received the fully filled pilot form. Initially, they had been told to assess the questionnaire by assigning a rating on a 5-point scale based on the relevance, importance, and applicability of each item. A higher score signifies a heightened degree of relevance, significance, or suitability ascribed to the item.

Afterwards, the content validity ratio (CVR) and content validity index (CVI) were calculated. 

In order to determine the content validity ratio, specialists were approached to gain their assessment regarding the requirement or lack thereof of each item. According to Lawshe’s table [72], the values of 0.99 were considered acceptable. Subsequently, the content validity index was computed by assessing the items according to their pertinence, lucidity, and straightforwardness, and only those with scores over 0.79 were deemed satisfactory.

Two questions were removed from the content validity index section based on expert evaluations, adhering to a predetermined threshold of 0.79. As a result, the 14 questions demonstrated appropriate content validity and progressed to the step of assessing construct validity. 

In the third phase, the questionnaire was given to a cohort of 20 persons who will not be included in the study. Their objective was to peruse the questionnaire, respond to the prompts, and bring up any problems or inquiries regarding the clarity or ambiguity of the questions.

Upon obtaining comments and ideas from the specified persons, we implemented the necessary revisions to improve the clarity of the items.

The minimum and maximum scores attainable in this 14-item evaluation were 14 and 70, correspondingly (see Table 1).

- Phase III: Structural Validation (1 May 2023–20 September 2023)

The study included a group of 454 individuals (156 men and 298 females) between the ages of 18 and 65. The primary criteria for inclusion were (i) a PCR-confirmed SARS-CoV-2 infection, (ii) a minimum interval of 6 months between the infection and participation in the research study, and (iii) obtaining written consent from individuals who are between the ages of 18 and 65 and ensuring they are fully informed about the study (Ethics Committee of University of Payame Noor Iran, Tehran, approved and permission for the study). The primary exclusion criterion was the presence of any pre-existing cognitive impairments or psychiatric disorders.

The recruitment process involved utilizing many avenues such as referrals, student groups, and online/social media platforms, notably the Telegram Long COVID Support Group, which boasts a membership of over 10,000 individuals.

The participants were administered the questionnaire using the online assessment tool “Porsline” [73].

After collecting data, the **Post-Covid Cognitive Impairment Scale** was validated using both exploratory factor analysis (EFA) and confirmatory factor analysis (CFA) methodologies.

### 2.3. Data Analysis

Descriptive statistics, such as frequency, percentage, mean, and standard deviation, were employed to depict the sociodemographic features of the collected sample. The **Post-Covid Cognitive Impairment Scale** was validated using both exploratory factor analysis (EFA) and confirmatory factor analysis (CFA) methodologies. In order to assess the suitability of the data for factor analysis, two indicators were employed: the Kaiser–Mayer–Oklin measure of sample adequacy (KMO) [74] and Bartlett’s test of sphericity [75]. The components were extracted using eigenvalue, scree plot, and Kaiser’s Rule [76], and the explained variance percentage was calculated. The analytical approach employed was maximum likelihood with direct oblimin [77]. Covariance-based structural equation modeling (CB-SEM) [78] and the robust maximum likelihood estimator technique (MLR) [79] were employed to conduct confirmatory factor analysis (CFA). The CFA analysis was performed using IBM SPSS Amos Graphic 26 [80]. The model fit assessment [81] indices used in this study included Chi-square (X2), normed chi-square (CMIN/DF), the Tucker and Lewis Index (TLI; with values ≥ 0.90), comparative fit index (CFI; with values ≥ 0.90), the standardized root mean square residual (SRMR; with values < 0.08), and the root mean square of approximation (RMSEA; with values ranging from 0.0 to 0.08). The reliability of the **Post-Covid Cognitive Impairment Scale** was evaluated by assessing its internal consistency through the use of Cronbach’s Alpha.

## 3. Results

### 3.1. Sample Characteristics

The study sample consisted of 454 patients. The age of participants spanned a wide range, from 18 to 65 years. Regarding sex, women comprised 65.6% of the participants, while men comprised the remaining 34.4%. According to educational attainment, 7.5% were under diploma, 12.6% were graduates, 33.5% had a bachelor’s degree, 34.1% had a master’s degree, and 12.3% had a doctoral degree. Regarding marital status, 28.2% of the participants were single, 66.1% were married, 1.5% were widowed, 3.3% were divorced females, and 0.9% were divorced males (see Table 2).

### 3.2. Validity

The results of EFA have been reported in Table 3, Table 4, Table 5 and Table 6. Table 3 shows the mean scores and standard deviations of the individual items.

Table 3 presents skewness and kurtosis data to provide a comprehensive analysis of the distribution shape for each item. Skewness denotes the asymmetry of responses relative to the mean, with values near zero indicating a more symmetric distribution. Positive skewness values suggest a distribution biased towards lower answer categories, whilst negative values show a bias towards higher categories. Kurtosis values denote the “tailedness” or peak of each item’s distribution, with values approaching zero signifying a shape akin to a normal distribution. Elevated positive kurtosis indicates a more pronounced peak, whereas negative values reflect a more subdued distribution. These tests assess each item’s conformity to normal distribution assumptions, thereby elucidating the scale’s psychometric characteristics.

The KMO index (0.89) and Bartlett’s test of sphericity (*p* < 0.001) indicate the suitability of the **Post-COVID Cognitive Impairment Scale** items for factor analysis (the data can be consulted in Table 4).

Two factors were extracted based on the eigenvalue, scree plot, and Kaiser’s Rule results. Also, the direct oblimin data showed a two-factor solution and a clear pattern. The results identified two factors that explained 40.38 of the total variances, with eigenvalues > 1 (Table 5). No items were removed from the scale. Seven items comprised one factor called “Memory.” Seven items comprised a second factor called “Attention”.

In order to gain a deeper understanding of the fundamental framework of the Post-COVID Cognitive Impairment Scale, a Pattern Matrix was created as a component of the exploratory factor analysis. This matrix offers a clear understanding of the distribution of each item among the specified components, indicating the level of correlation between each item and a particular component. The factor loadings of the 14 items over two unique components, namely memory and attention, are presented in Table 6. Items that have higher loadings on a specific component suggest a more pronounced association with that cognitive domain, showcasing the scale’s capacity to accurately assess these diverse elements of cognitive function.

In Table 6. factor loadings exceeding 0.4 are deemed significant, as they signify a considerable link between the item and the respective factor. Loadings beneath this threshold (0.4) are deemed negligible and so are not emphasized as principal markers of factor structure. Furthermore, cells that are unfilled in the table signify factor loadings below 0.1, which were omitted for clarity and to emphasize the most pertinent item-factor associations.

Furthermore, CFA was conducted to verify the cognitive functions scale. The results of CFA indicate that the values of all indices are desirable. In other words, the fit indices of the model indicate the desirability of the **Post-Covid Cognitive Impairment Scale** measurement model (the data can be consulted in Table 7).

The values of factor loadings (Table 8) illustrate that the cognitive functions scale is valid at the items level.

### 3.3. Reliability

The reliability assessment included an evaluation of internal consistency. Cronbach’s alpha coefficient for the memory factor was 0.81; the attention factor was 0.80. As a result, the **Post-Covid Cognitive Impairment Scale** is reliable; it has internal consistency and measurement precision.

## 4. Discussion

### 4.1. Main Findings

The objective of this study was to create and validate the Post-COVID Cognitive Impairment Scale, an innovative instrument intended to evaluate the seriousness of cognitive impairment symptoms in persons who have long COVID. This study’s findings offer valuable insights into the scale’s reliability and validity, emphasizing its potential usefulness in clinical and research environments.

The Post-COVID Cognitive Impairment Scale exhibited robust psychometric characteristics, affirming its accuracy and consistency in assessing cognitive deficits across two specific areas: memory and attention. Both the exploratory factor analysis (EFA) and confirmatory factor analysis (CFA) provided evidence in favor of the scale’s two-component structure, which accounted for 40.38% of the total variance. The KMO measure of sample adequacy (0.89) and Bartlett’s test of sphericity (*p* < 0.001) have verified that the data is suitable for factor analysis, suggesting a strong component structure.

The CFA findings provided additional evidence supporting the reliability of the scale, as indicated by the fit indices such as CMIN/DF (2.80), RMSEA (0.06), SRMR (0.05), CFI (0.93), and TLI (0.92), all of which suggest a strong match. Furthermore, the factor loadings for each item were statistically significant, which strengthens the evidence supporting the scale’s construct validity. The scale’s internal consistency was verified using Cronbach’s alpha coefficients. The coefficients for memory and attention were 0.81 and 0.80, respectively, indicating reliable performance ranging from acceptable to good.

### 4.2. Comparative Analysis with Prior Studies

This study’s findings align with an expanding corpus of literature emphasizing the cognitive deficits linked to long COVID, often known as neuro-long COVID [11,17,35,41]. Consistent with other studies, our findings highlight substantial impairments in memory and attention in individuals who have recovered from COVID-19, including those with moderate acute symptoms who did not necessitate hospitalization [29,30,31]. This corresponds with research indicating that cognitive impairments are widespread across multiple domains, such as memory, attention, executive function, and problem-solving skills [11,12,42,43,61,62,65,66].

Prior neuropsychological evaluations have consistently revealed cognitive impairments in survivors of COVID-19 [61,62,64]. Almeria et al. [61] and Becker et al. [62] employed extensive neuropsychological test batteries to identify abnormalities in attention, executive functioning, and memory. Although these assessments offer comprehensive insights, their applicability is constrained by the requirement for specialist staff and prolonged administration time [42]. This study contributes to the field by presenting the Post-COVID Cognitive Impairment Scale, a proven and effective tool specifically created to evaluate cognitive abnormalities in post-COVID individuals. This instrument provides a practical option that can be easily utilized in clinical and research environments.

Unlike previous evaluation instruments that frequently combine somatic, psychological, and cognitive symptoms, such as the Symptom Burden Questionnaire by Hughes et al. [45] and the scale by Bahmer et al. [46], our instrument is solely dedicated to cognitive deficits. The Symptom Burden Questionnaire, although extensive with 131 items over 17 categories, is unwieldy for regular application. Bahmer et al.’s scale predominantly focuses on physical sequelae and necessitates intricate analytical techniques, hence constraining its utility for cognitive evaluation. By focusing on memory and attention, our scale meets the essential requirement for a targeted assessment of cognitive processes that directly influence daily living and quality of life in patients with long COVID.

Furthermore, our research addresses the recognized deficiency in the availability of particular, validated questionnaires for post-COVID cognitive impairment [11,17,44]. Although instruments such as the Neurobehavioral Symptom Inventory (NSI) [50] and the Symptom Burden Questionnaire for Long COVID (SBQ-LC) [45] encompass cognitive symptoms, they fail to offer the requisite granularity for comprehensive cognitive evaluation and may lack sensitivity to nuanced deficits encountered by patients. The NSI includes somatosensory, cognitive, and affective symptoms but may not adequately reflect specific cognitive difficulties, such as memory deficiencies and attentional lapses, commonly observed in long COVID [51,52].

Our findings corroborate research indicating the prevalence of cognitive symptoms in patients with long COVID, irrespective of the original infection’s severity [18,19,29,30,31]. This highlights the imperative for extensive cognitive assessment utilizing accessible instruments such as our scale. The significance of patient-reported outcome measures (PROMs) is clear since they offer critical insights into the impact of cognitive deficits on patients’ daily functioning [57,58]. Nonetheless, dependence on self-reported data may introduce biases, a drawback acknowledged by our work, which is addressed by stringent validation procedures, including exploratory and confirmatory factor analyses.

Moreover, our research enhances the discussion over the ecological validity of cognitive evaluations in long COVID. Conventional neuropsychological assessments, while comprehensive, may not accurately represent patients’ cognitive experiences in real-world contexts [17,44,70]. Our scale provides an ecologically valid assessment by creating a questionnaire that reflects patients’ daily experiences with memory and attention, so addressing the dynamic and situational aspects of cognitive impairments in long COVID.

Although our scale provides a useful instrument for the preliminary assessment of cognitive impairments in patients with long COVID, it is crucial to recognize its limits. This questionnaire is intended as a first step in identifying persons who may be experiencing cognitive impairments. Additional extensive neuropsychological assessments that specifically evaluate cognitive functioning are advised to confirm diagnoses and formulate thorough intervention strategies. This approach corresponds with previous research highlighting the necessity for comprehensive evaluations after initial screenings to thoroughly comprehend the severity of cognitive impairments in persons with long COVID-19 [61,62,64].

### 4.3. Clinical Practice Consequences

The validated Post-COVID Cognitive Impairment Scale has important implications for clinical practice. It offers healthcare providers a dependable and accurate instrument for evaluating cognitive deficits in persons who have long COVID, making it easier to detect and address these issues at an early stage. The scale may be utilized to observe the advancement of symptoms and the effectiveness of treatment, consequently enhancing patient results.

Utilizing this scale to identify cognitive deficits can also provide valuable insights for the creation of focused rehabilitation programs. Comprehensive treatment, which encompasses cognitive rehabilitation and mental health assistance, can be customized to meet the unique requirements of persons suffering from extended COVID, therefore improving their quality of life and functioning capabilities. 

Moreover, the emphasis of the scale on cognitive impairments underscores the significance of addressing mental health in addition to physical health in post-COVID treatment. Adopting a holistic approach is crucial for effectively managing and facilitating the complete healing of patients.

### 4.4. Limitations and Prospects for Further Investigation

Although this study offers strong evidence supporting the validity and reliability of the Post-COVID Cognitive Impairment Scale, it is important to recognize several limitations. The cross-sectional approach hampers the capacity to evaluate alterations in cognitive deficits over a period of time. In order to monitor the development of cognitive symptoms and evaluate the impact of therapies, it is recommended that future studies utilize longitudinal designs.

Furthermore, the study sample was specifically selected from the geographic region of Iran, which might potentially restrict the applicability of the results to a broader population. It is necessary to conduct replication studies in various communities and situations to validate the scale’s suitability for different cultural and demographic groupings.

Further investigation is required to examine the correlation between cognitive deficits and additional long-term symptoms of COVID-19, including tiredness and respiratory problems. Gaining insight into these interconnections can offer a more thorough understanding of the post-COVID state and guide complete treatment strategies.

Additionally, the utilization of an online platform (e.g., Porsline) for data gathering in the validation of the cognitive assessment tool entails some limitations. Online platforms provide considerable benefits regarding accessibility and ease, allowing efficient engagement with a wide and varied participant base; nevertheless, they also present certain restrictions that may affect data dependability and validity. Online evaluations do not provide the controlled atmosphere of in-person settings, perhaps leading to distractions or inconsistencies in participant engagement with the questionnaire. The absence of control may impact the uniformity of responses, particularly in self-reported cognitive evaluations when external variables can impair focus and comprehension. Moreover, participants’ proficiency with digital devices may differ; those with restricted digital literacy can encounter challenges in navigating the online platform, perhaps leading to biases or compromising the accuracy of their responses. Moreover, online data collecting may unintentionally exclude persons with significant cognitive impairments or those lacking dependable internet connection, thereby compromising the generalizability of the results. To improve reliability, future validations may utilize a hybrid approach that integrates online and in-person data collection methods, thus enhancing data quality and inclusivity.

In addition, although the Post-COVID Cognitive Impairment Scale serves as a useful instrument for the preliminary assessment of cognitive deficits, it is crucial to acknowledge its limitations when used alone. The questionnaire is intended as a first step in identifying individuals who may be experiencing cognitive impairments. Consequently, more thorough neuropsychological assessments that specifically evaluate cognitive functioning are advised to validate diagnoses and formulate complete remediation strategies. Integrating objective cognitive evaluations with the scale can improve diagnostic precision, offer a deeper insight into the severity and characteristics of cognitive deficits, and guide more targeted and effective treatment approaches.

## 5. Conclusions

The Post-COVID Cognitive Impairment Scale is an accurate and dependable tool for evaluating cognitive deficits in persons who are recuperating from COVID-19. This instrument fulfills an important requirement by providing a realistic and targeted method to assess cognitive impairments in this specific group. The scale’s strong psychometric qualities validate its use in clinical and research environments, enabling the early detection, tracking, and treatment of cognitive deficits linked to long COVID.

The Post-COVID Cognitive Impairment Scale offers a consistent approach to evaluating cognitive impairments. Its use can enhance patient treatment and results, aiding in the continuous endeavors to tackle the lasting effects of COVID-19. Subsequent investigations should further improve and authenticate this instrument, guaranteeing its pertinence and efficiency in various demographics and environments.

## Figures and Tables

**Table 1:** Post-Covid Cognitive Impairment Scale.

Items	Questions
V1	The extent of your difficulty in remembering tasks or activities you intend to perform.
V2	The extent of your difficulty in recalling events that occurred to you in the past week.
V3	The extent of your difficulty in remembering the names of individuals you interact with daily.
V4	The extent of your difficulty in recognizing individuals you have previously met.
V5	The extent of your difficulty in remembering the reason for leaving your house.
V6	The extent of your difficulty during conversations: forgetting the topic of discussion and going off track.
V7	The extent of your difficulty in finding items because you placed them in the wrong location and cannot remember where.
V8	The extent of your difficulty in effectively learning new skills.
V9	The extent of your difficulty in maintaining focus due to minor distractions and ambient noise.
V10	The extent of your difficulty in fully assessing situations when making decisions.
V11	The extent of your difficulty in distinguishing between important and unimportant aspects while performing a task.
V12	The extent of your difficulty in listening attentively and without distraction to a lecture.
V13	The extent of your difficulty in concentrating on studying a single topic for more than ten minutes.
V14	The extent of your difficulty in taking notes while simultaneously listening to a lecture.

Each item in the Post-COVID Cognitive Impairment Scale was rated by participants on a five-point Likert scale, where 1 = Very Little, 2 = Little, 3 = Moderate, 4 = Much, and 5 = Very Much, with higher scores indicating greater impairment.

**Table 2:** Characteristics of study sample (n = 454).

Characteristic	N	%
**Sex**		
Female	298	65.6
Male	156	34.4
**Education**		
Under diploma	34	7.5
High School Diploma	57	12.6
Bachelor’s Degree	152	33.5
Master’s Degree	155	34.1
Doctoral Degree	56	12.3
**Marital status**		
Single	128	28.2
Married	300	66.1
Widow	7	1.5
Divorce-Female	15	3.3
Divorce-Male	4	0.9

**Table 3:** Items analysis of Post-Covid Cognitive Impairment Scale.

Items	Mean	SD	Skewness	Kurtosis
V1	2.36	1.04	0.43	−0.32
V2	2.17	1.09	0.63	−0.41
V3	1.75	1.02	1.24	0.75
V4	1.91	0.99	0.97	0.40
V5	1.35	0.65	1.62	1.42
V6	1.80	0.92	1.13	1.06
V7	2.44	1.11	0.60	−0.22
V8	2.28	1.11	0.50	−0.59
V9	2.51	1.16	0.40	−0.67
V10	2.03	1.07	0.86	−0.03
V11	1.86	0.94	1.08	0.94
V12	3.31	1.29	−0.23	−1.06
V13	2.46	1.17	0.48	−0.55
V14	2.23	1.21	0.75	−0.46

**Table 4:** Values estimation of KMO and Bartlett’s Test.

KMO and Bartlett’s Test		
Kaiser–Meyer–Olkin Measure of Sampling Adequacy.		0.895
Bartlett’s Test of Sphericity	Approx. Chi-Square	2059.983
	df	91
	Sig.	0.000

**Table 5:** Rotation sums of squared loadings.

Component	Rotation Sums of Squared Loadings		
	Total	% of Variance	Cumulative %
Memory	4.71	33.63	33.63
Attention	0.95	6.75	40.38

**Table 6:** Pattern Matrix.

Items	Component	
	1	2
**V1**	0.625	
**V2**	0.752	
**V3**	0.630	
**V4**	0.563	
**V5**	0.461	0.134
**V6**	0.515	0.256
**V7**	0.448	0.273
**V8**	0.214	0.486
**V9**	0.161	0.530
**V10**		0.803
**V11**		0.716
**V12**	0.183	0.412
**V13**	0.193	0.573
**V14**	0.173	0.415

**Table 7:** Values estimation of confirmatory factor analysis indexes (CB-SEM).

Indices	CFA Index Standard	Model
Chi-square	-	210.20
DF	-	75
Normed chi-square (CMIN/DF)	<5	2.80
RMSEA	<0.08	0.06
SRMR	<0.08	0.05
CFI	≥0.90	0.93
TLI	≥0.90	0.92

**Table 8:** Factor loading of Post-Covid Cognitive Impairment Scale.

Component	Items	Factor Loading *	Critical Ratio *	*p*. Value *
**Memory**	V1	0.67	13.21	0.001
	V2	0.74	13.21	0.001
	V3	0.56	10.48	0.001
	V4	0.47	8.92	0.001
	V5	0.56	10.39	0.001
	V6	0.70	12.67	0.001
	V7	0.64	11.88	0.001
**Attention**	V8	0.65	11.22	0.001
	V9	0.64	11.22	0.001
	V10	0.54	9.72	0.001
	V11	0.55	9.91	0.001
	V12	0.57	10.23	0.001
	V13	0.73	12.35	0.001
	V14	0.55	9.27	0.001

* “Factor Loading” refers to the strength of the relationship between each item and the underlying factor. “CR” (Critical Ratio) indicates the standardized test statistic for each item loading, which tests the significance of the loading. “*p*-value” represents the statistical significance level for each loading, with values below 0.05 indicating that the factor loading is statistically significant.

## Data Availability

Data that substantiate the conclusions of this investigation are accessible from the corresponding author upon reasonable request.
